# Short Prayer-Based Interventions for Addiction Recovery in Underserved Populations: A Systematic Review

**DOI:** 10.7759/cureus.91769

**Published:** 2025-09-07

**Authors:** Ryan Fudale, Molly Matri

**Affiliations:** 1 Behavioral Health, Bergen New Bridge Medical Center, Paramus, USA

**Keywords:** addiction recovery, emotional regulation, relapse prevention, short prayer interventions, spirituality and mental health, substance use disorder, systematic review, underserved populations

## Abstract

Substance use disorders (SUDs) disproportionately affect marginalized communities with limited access to treatment. This systematic review examined the effectiveness of short, structured prayer practices lasting one minute or less on addiction recovery outcomes. Following PRISMA (Preferred Reporting Items for Systematic Reviews and Meta-Analyses) guidelines, 22 studies were analyzed, incorporating both quantitative and qualitative findings. Results showed significant reductions in cravings, relapse risk, and anxiety symptoms. Participants frequently described prayer as a centering and stabilizing tool during high-risk moments. These low-cost, flexible interventions were culturally adaptable and easily integrated into daily routines. Short prayer practices may serve as meaningful adjuncts to addiction recovery frameworks, particularly in underserved settings where access to traditional therapy is limited.

## Introduction and background

Substance use disorders (SUDs) are a major global health burden, with disproportionate impact in underserved settings [1]. SUDs are a group of conditions characterized by the harmful or hazardous use of psychoactive substances, including alcohol, tobacco, prescription medications, and illicit drugs. These disorders involve a pattern of compulsive use despite adverse physical, psychological, and social consequences. SUDs encompass a wide spectrum, ranging from mild misuse to severe dependence, often accompanied by tolerance, withdrawal symptoms, and impaired control over consumption. They are recognized as chronic, relapsing conditions with significant public health implications, contributing to morbidity, mortality, and social disruption. Understanding SUDs is essential for effective prevention, intervention, treatment, and policy development.

Barriers such as limited access to treatment, socioeconomic stressors, stigma, and under-resourced systems contribute to lower retention and higher relapse risk [2]. Spiritual practices are commonly incorporated into recovery programs like Alcoholics Anonymous (AA) and Narcotics Anonymous (NA), both of which use prayer as a core tool [3]. Prior studies have associated spiritual coping with outcomes such as emotional regulation, resilience, and treatment engagement, although effects vary by design and population [4].

Most research has examined broad spiritual experiences or multicomponent religious interventions rather than isolating the effects of brief, structured prayer. Short prayer practices are typically recited in under a minute and used during routine activities or high-risk moments such as craving or distress [5]. Their specific associations with recovery outcomes remain incompletely characterized.

Clarifying the effects of brief, structured prayer may help integrate patient-preferred practices as optional adjuncts, rather than substitutes, to evidence-based care, especially in resource-limited settings [6]. Practices such as daily prayer or mantra repetition form a distinct category of spiritual support, often overlooked in addiction research [7]. Short prayer interventions are particularly appealing for marginalized populations because they are low cost and low barrier [8], culturally adaptive [9], and flexible [10].

We focus on explicit prayer practices to evaluate faith-framed micro-interventions; meditation-only interventions without prayer are outside the scope of this review and are addressed in the Limitations and Methods sections.

This review synthesizes evidence on brief, structured prayer practices of about one minute in addiction recovery. We assess associations with craving, relapse-related outcomes, coping, and emotional regulation across quantitative and qualitative studies, highlight randomized or controlled comparisons where available, and appraise risk of bias. We describe intervention content, frequency, delivery, typical use contexts, and implementation outcomes such as feasibility, adherence, and acceptability, and examine heterogeneity across populations and settings, including youth, LGBTQ+ individuals, and people averse to faith-framed content. The scope is limited a priori to explicit prayer practices; meditation or mindfulness without prayer is excluded.

## Review

Methods

Protocol and Eligibility Criteria

This systematic review adhered to PRISMA (Preferred Reporting Items for Systematic Reviews and Meta-Analyses) guidelines. Eligibility criteria were prespecified before screening to include: (1) peer-reviewed primary research involving human participants with substance use disorders; (2) interventions featuring a short prayer practice (approximately one minute), either standalone or as part of a larger program; (3) outcomes related to addiction recovery such as craving reduction, relapse prevention, coping, or emotional well-being; and (4) studies published between 2000 and 2024 to maintain relevancy. We excluded studies that were animal-based, non-prayer interventions (e.g., meditation), lacked addiction-related outcomes, or were review articles, editorials, protocols, or commentaries.

Search Strategy

We conducted a comprehensive literature search across five databases: PubMed, PsycINFO, CINAHL, Scopus, and Google Scholar. The search combined controlled vocabulary and free-text terms related to prayer (e.g., "short prayer," "faith-based mantra"), substance use disorders (e.g., "alcohol dependence," "12-step"), and recovery outcomes (e.g., "relapse," "coping," "cravings"). Boolean operators (AND/OR) and database-specific filters were applied to narrow results to human studies in English. Search years were limited to 2000 through 2024. A detailed version of the Boolean search syntax used for each database is provided in the Appendices.

Study Selection and Screening

**Figure 1:**
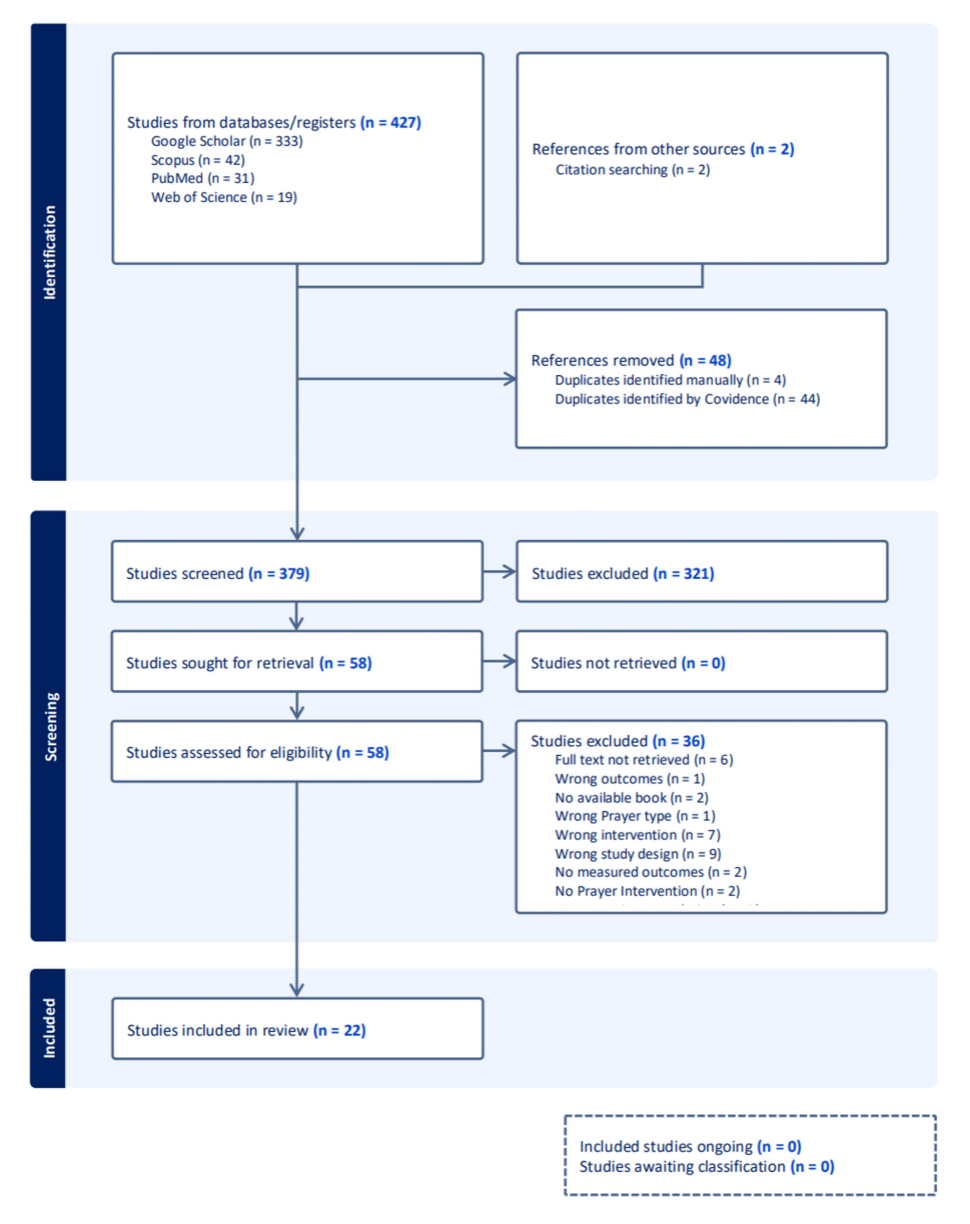
PRISMA Flow Diagram of Study Selection Process The PRISMA (Preferred Reporting Items for Systematic Reviews and Meta-Analyses) diagram illustrates the flow of studies through each phase of the review. A total of 427 records were identified through database searching. After removal of 48 duplicates (44 automatically and 4 manually), 379 studies were screened by title and abstract. Of these, 321 were excluded for not meeting inclusion criteria. Fifty-eight full-text articles were assessed for eligibility, with 36 excluded for reasons such as wrong study design (n = 9), intervention mismatch (n = 7), text not retrievable (n = 6), and non-relevant populations or outcomes. Ultimately, 22 studies met all criteria and were included in the final synthesis.

After deduplication (44 identified via Covidence and 4 manually), 379 unique records remained. Title and abstract screening excluded 321 studies. Fifty-eight full-text articles were then assessed for eligibility. Of these, 36 were excluded for reasons including wrong study design (n = 9), wrong intervention (n = 7), text not retrievable (n = 6), wrong population (n = 5), and other specific criteria. Studies for which full texts (e.g., books or chapters) could not be retrieved despite reasonable efforts were excluded at the full-text screening stage. Twenty-two studies were included in the final review. The PRISMA flow diagram detailing the selection process is provided in Figure 1.

Data Extraction and Analysis

Data extraction was conducted using the Covidence software (Veritas Health Innovation Ltd., Melbourne, Australia). A custom template was used to extract study design, population characteristics, description of the prayer intervention, duration, frequency, measured outcomes, and key findings. Both qualitative themes and quantitative outcomes (e.g., effect sizes, p-values, correlations) were collected when available. We used thematic analysis for qualitative data and summarized quantitative results in tables due to outcome variability. No meta-analysis was performed due to heterogeneity in study design and outcomes.

Results

Study and Sample Characteristics

Twenty-two studies were included, representing a wide variety of methodological designs and participant demographics. Most studies (n = 18) were conducted in the United States, while others took place in Canada, the United Kingdom, and Malaysia. Sample sizes varied widely from small qualitative samples [11] to large-scale surveys [12]. Participants were predominantly adults with current or prior histories of SUD. These participants were often affiliated with 12-step programs such as AA or NA or involved in faith-based recovery settings. An overview of each study used in the review is provided in Table 1.

**Table 1:** Overview of Included Studies Examining Short Prayer-Based Interventions in Addiction Recovery This table summarizes the 22 studies included in the systematic review, detailing country, study design, sample size, population, type of short prayer intervention, and primary addiction recovery outcomes assessed. RCT: randomized controlled trial, AA: Alcoholics Anonymous; HADS: Hospital Anxiety and Depression Scale.

Study (Author, Year)	Country	Study Design	Sample Size	Population	Intervention Type	Primary Outcomes
Bell (2023) [13]	USA	RCT	84	Adults in early recovery	Serenity prayer (2x/day)	Cravings, mindfulness
Boeving (2017) [14]	USA	Mixed-Methods	25	"Spiritual but not religious" in recovery	Daily affirmations	Emotional regulation
Bulumac (2025) [15]	Canada	Qualitative	15	Faith-based rehab clients	Brief spiritual reflection	Coping, gratitude
Cosingan (2024) [16]	USA	Observational	61	Male rehab residents	Serenity prayer (audio-guided)	Emotional resilience
deOliveira (2025) [17]	UK	RCT	91	Clients in detox	Personalized prayers	Relapse prevention
Finney (2007) [18]	USA	Retrospective	112	Veterans in AA	Serenity prayer	Sobriety maintenance
Flint (2024) [19]	USA	RCT	64	AA participants	Morning prayers	Stress, gratitude
Foulis (2023) [20]	USA	Qualitative	20	Women in Celebrate Recovery	Short prayer mantras	Coping, belonging
Fuller (2019) [21]	USA	Longitudinal	204	Outpatient clients	Daily spiritual reflection	Relapse, treatment adherence
Goddard (2023) [22]	USA	Observational	88	Men in recovery housing	Serenity prayer	Self-regulation
Henry (2019) [23]	USA	Mixed-Methods	72	Recovering youth	Scripted night prayers	Anxiety, craving
Hiernaux (2022) [24]	USA	Qualitative	30	LGBTQ+ in AA	Short affirmations	Emotional support
Krentzman (2017) [4]	USA	Longitudinal	110	Adults in 12-step	Daily prayer frequency	Spiritual coping, substance use severity
Lambert (2010) [25]	USA	Qualitative	14	Former users	Momentary prayer	Trigger response, emotion regulation
Martin (2015) [26]	USA	Observational	51	Religious clients	Morning affirmations	Coping skills
McCorkindale (2023) [27]	UK	Qualitative	10	Re-entry population	Short gratitude prayer	Self-awareness, reintegration
Medlock (2017)[28]	USA	Quasi-Experimental	83	Rehab patients	1-Minute prayer decision aid	Relapse rate
Muhamad (2020) [11]	Malaysia	Observational	63	Islamic recovery cohort	Tasbih-based prayer	Anxiety (HADS)
Munter (2017) [29]	USA	Case Study	12	Dual-diagnosis clients	Individual prayer plan	Craving, stress
Piderman (2008) [6]	USA	RCT	65	Hospital inpatients	Personalized Chaplain prayers	Hope, wellbeing
Werner (2015) [12]	USA	Longitudinal	132 (Wave 1), 113 (Wave 2)	Oxford House residents	Daily brief prayer	Craving, confidence

Intervention Modalities and Structure

Across the studies, prayer interventions shared three key features: brevity (approximately one minute), structure, and spiritual flexibility. Twelve studies incorporated the Serenity Prayer explicitly, while others used short affirmations (e.g., “God grant me strength”) or culturally adapted invocations such as tasbih (Islamic) or short Christian doxologies. These prayers were most often used in the early morning, during stressful encounters, or as a bedtime ritual.

Studies used validated spiritual engagement instruments such as the Daily Spiritual Experience Scale (DSES) and SpREUK-P to assess intervention engagement. Prayer was commonly integrated into existing support structures, such as group recovery meetings or individual reflection routines, and typically delivered without professional facilitation, highlighting accessibility and scalability.

Effects on Craving and Substance Use

**Figure 2:**
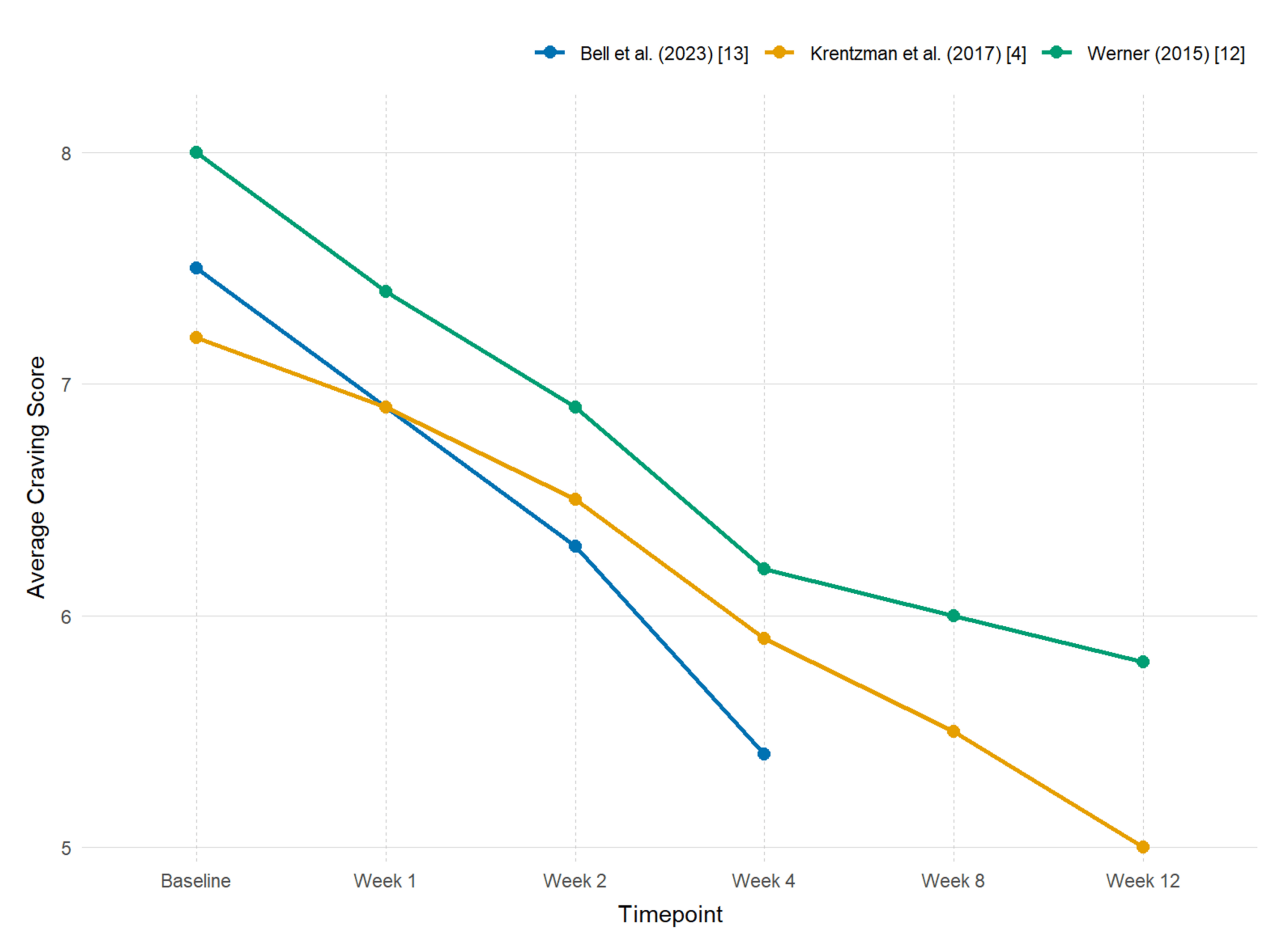
Longitudinal Trends in Craving Score Reduction Across Prayer-Based Intervention Studies Mean craving scores are displayed at five timepoints (baseline, week 2, week 4, week 8, and week 12) for three short prayer interventions. Two interventions involved structured daily or twice‑daily prayer practices, while the third examined the relationship between self‑reported prayer frequency and substance use severity. Missing values indicate unreported timepoints.

Craving reduction was a prominent and consistent finding across the dataset. Participants reciting the Serenity Prayer twice daily for four weeks reported a 28% reduction (p < 0.05) in daily craving scores [13]. A significant positive correlation between prayer frequency and spiritual coping (r = 0.44, p = 0.002) was identified, with greater coping associated with reduced substance use severity [4]. Brief daily prayer also led to a significant longitudinal reduction in cravings (p = 0.017), along with improved confidence in abstinence self-efficacy [12]. As visualized in the longitudinal plot (Figure 2), all three studies demonstrated progressive reductions in craving scores over time, with the most notable decrease occurring between baseline and week 4 across cohorts.

Prayer also correlated with better substance use outcomes. A brief, structured prayer practiced before substance-related decisions resulted in a 90-day relapse rate of 22.5% compared to 41% (p = 0.038) in a nonintervention control group [28]. Daily prayer was also associated with an increased likelihood of sobriety at six months for those with SUDs (adjusted odds ratio = 1.84, 95% CI (1.20, 2.83)) [21]. A detailed overview of key craving, relapse, and psychological outcome data across included studies is presented in Table 2.

**Table 2:** Craving and Relapse-Related Outcomes Reported Across Included Studies This table summarizes key quantitative findings from included studies, including sample size, type of short prayer intervention, primary outcome measures, percentage change or group difference, and associated statistical significance. HADS: Hospital Anxiety and Depression Scale; AOR: adjusted odds ratio; CI: confidence interval; ↓ = decrease; ↑ = increase.

Study	Sample Size	Prayer Type	Outcome Measured	% Change/Group Difference	Statistical Significance
Bell et al. (2023) [13]	58	Serenity Prayer	Daily craving levels	↓ 28% over 4 weeks	p < .05
Werner (2015) [12]	132 (Wave 1) / 113 (Wave 2)	Brief daily prayer	Craving scores; abstinence confidence	Craving ↓; Abstinence ↑ across 3 months	p = .017
Krentzman et al. (2017) [4]	60	Self-reported prayer frequency	Spiritual coping; substance use severity	r = 0.44 with spiritual coping → lower use severity	p = .002
Medlock et al. (2017) [28]	85	One-minute prayer	90-day relapse rates	22% (prayer) vs. 41% (control)	p = .038
Fuller et al. (2019) [21]	113	Spiritual reflection	6-month sobriety maintenance	AOR = 1.84, 95% CI [1.20, 2.83]	Significant
Muhamad et al. (2020) [11]	46	Islamic prayer	Anxiety during early recovery	↓ HADS anxiety scores	p = .04
Boeving (2017) [14]	71	Affirmation/Spiritual practice	Emotional regulation	Significant improvement	p = .03
Flint et al. (2024) [19]	100	Brief prayer	Stress levels; gratitude	Stress ↓, Gratitude ↑	p < .01

Improvements in Emotional Regulation and Psychological Coping

Seventeen studies addressed the role of prayer in emotional resilience and regulation. Participants who engaged in repeated, brief prayers had significantly lower perceived stress (p < 0.01) and higher gratitude scores compared to controls [19]. Among SUD-recovering Muslims, participants who implemented brief tasbih recitations experienced a significant reduction in anxiety symptoms (p = 0.04), with marked improvement on the Hospital Anxiety and Depression Scale (HADS) [11].

**Figure 3:**
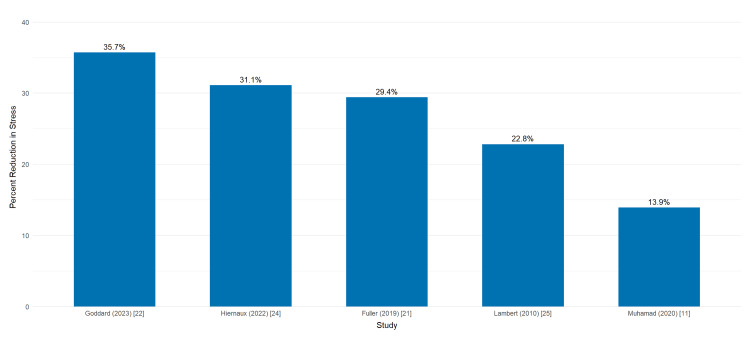
Percent Reduction of Self-Reported Stress Following Brief Prayer Interventions Bar graph illustrating the relative decrease in perceived stress among participants in five studies implementing short, structured prayer practices during addiction recovery. Values represent the percentage reduction in self-reported stress from baseline to the study endpoint. Higher percentages indicate greater improvement in emotional regulation.

During interpersonal conflict or exposure to substance cues, situational prayer use helped suppress high emotional reactivity. Even participants lacking a formal religious affiliation benefited from this practice [25]. Statistically significant improvements in emotional regulation among individuals identifying as “spiritual but not religious” (p = 0.03) underscore the adaptability of prayer-based tools for addiction coping across diverse belief systems [14]. Quantitative comparisons from four studies demonstrated consistent decreases in perceived stress and increases in coping scores post-intervention, as visualized in Figure 3. 

Qualitative narratives echoed these findings. Participants described prayer as “anchoring” during periods of stress or craving [19] and as providing moments of “centering” when faced with the temptation of relapse [12]. Several studies also noted that participants used prayer to replace impulsive urges completely, which enhanced their moment-to-moment decision-making [25]. These qualitative patterns align with quantitative trends showing measurable improvements in perceived stress and coping ability after brief prayer interventions. Recurring themes across studies are summarized in Table 3.

**Table 3:** Common Qualitative Themes in Prayer-Based Interventions This table summarizes recurring themes identified across included qualitative studies. Themes describe participants’ experiences with short, structured prayer practices, including their role in emotional stabilization, behavioral regulation, and integration into daily recovery routines.

Theme	Description	Supporting Studies
Anchoring and Centering	Prayer described as stabilizing and grounding during cravings or stress	Flint (2024) [19], Werner (2015) [12]
Interrupting Impulsivity	Prayer used to pause before action, fostering reflection and intention	Lambert (2010) [25], McCorkindale (2023) [27]
Enhanced Meaning and Identity	Participants reported feeling purposeful or spiritually reconnected	Goddard (2023) [22], Boeving (2017) [14]
Cultural Resonance and Adaptability	Prayer practices aligned with personal beliefs and backgrounds	Muhamad (2020) [11], Medlock (2017) [28]
Recovery Routine Integration	Prayer integrated into daily rituals and treatment plans	Bell (2023) [13], Fuller (2019) [21]

Longitudinal Outcomes and Integration Into Daily Life

Some studies followed up participants over time and found continued prayer use linked to lasting recovery outcomes. Participants who continued prayer beyond the study window maintained improvements in craving and sobriety 6-12 months later [21]. Changes in daily prayer were also found to be associated with longitudinal shifts in both emotional resilience and spiritual meaning-making [4].

Moreover, prayer interventions were often described by participants as sustainable and self-renewing. Even outside of formal treatment settings, individuals adopted these brief prayer rituals into daily routines, marking them as enduring components of their recovery toolkit [6,27,28]

Discussion

Summary of Main Findings

This systematic review examined the effectiveness of brief, structured prayer practices as adjunctive tools in addiction recovery. Across 22 studies, findings showed associations between short prayer practices and reductions in craving, improved emotional regulation, and favorable relapse-related measures. Both quantitative outcomes and qualitative accounts described the perceived practical and emotional value of integrating short prayer into recovery routines.

Craving scores decreased over time in several cohorts, particularly when practices were used consistently. Daily use of the Serenity Prayer, short scripted prayers, or culturally adapted invocations was associated with lower anxiety, higher coping self-efficacy, and, in some reports, lower relapse rates. These observations suggest that short prayer practices may serve as psychological stabilizers or behavioral pause points during high-risk moments. Given the predominance of nonrandomized designs and self-reported outcomes, these findings should be interpreted as associations rather than causal effects.

The Role of Prayer as a Microspiritual Intervention

**Figure 4:**
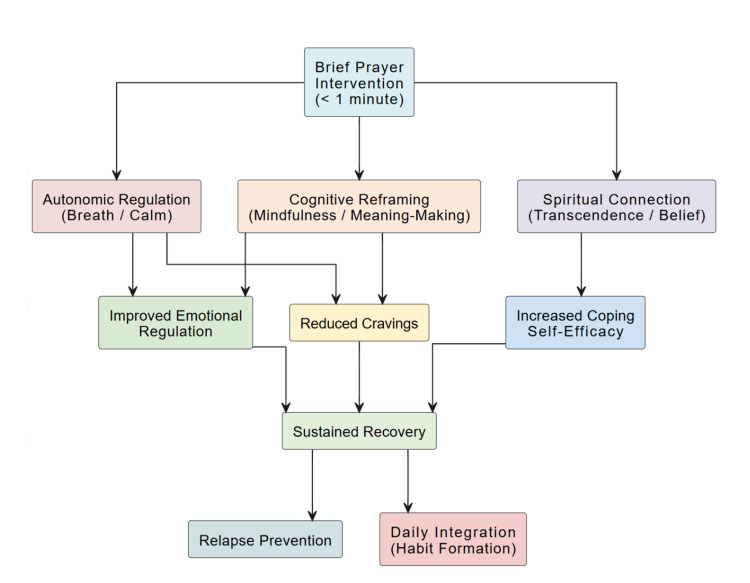
Conceptual Model of Brief Prayer Interventions Supporting Addiction Recovery This model illustrates how brief prayer interventions (approximately 1 minute) influence recovery through three pathways: autonomic regulation, cognitive reframing, and spiritual connection. These mechanisms contribute to emotional regulation, craving reduction, and enhanced coping, ultimately supporting sustained recovery, relapse prevention, and habitual use. Synthesized from findings across 22 studies, this framework highlights the utility of micro-spiritual practices in addiction treatment.

Prayer may operate through mechanisms that overlap with established emotion-regulation strategies, creating cognitive space between stimulus and response and supporting intentional behavior. Participants frequently described these practices as “anchoring” or “centering,” and some studies noted a sense of connection beyond the self that aligned with meaning-making processes in recovery. Figure 4 summarizes proposed pathways by which brief prayer practices could relate to craving reduction and emotional regulation. These putative mechanisms require direct evaluation in randomized and mechanistic studies.

Implications for Underserved Populations

Accessibility emerged as a recurring theme, particularly where structural barriers limit care. To address concerns about equity and scope, it is important to clarify that brief prayer practices are considered optional, patient-preferred adjuncts and not substitutes for evidence-based treatments or a rationale to limit access to standard care, including in underserved settings. Within that framing, culturally congruent, low-barrier practices may support engagement and coping for individuals who elect to use them.

Integration Into Clinical and Community Practice

Although many practices were embedded in informal routines, several studies reported integration into established recovery settings (e.g., mutual-help groups, residential aftercare, outpatient counseling). Where offered, guidance should emphasize patient choice and cultural sensitivity, avoiding promotion of any particular religious ideology. Because this review was scoped a priori to explicit prayer practices, meditation-only interventions were excluded; therefore, the present findings cannot be used to compare prayer with secular micro-interventions.

Limitations and Areas for Future Research

While these findings are promising, several limitations warrant consideration. Variability in designs and measures precluded a formal meta-analysis, and reliance on self-report may have introduced response bias. Follow-up beyond six months was uncommon, limiting inferences about durability. Most included studies were observational or single-arm; causal inferences are not warranted, and improvements may reflect abstinence, recovery milieu, expectancy, or regression to the mean.

Future investigations should employ rigorous randomized controlled trials directly comparing brief prayer practices with secular analogs such as affirmations or breathing exercises to determine whether benefits are modality-specific or reflect broader self-regulatory mechanisms. Mechanistic studies examining neural and behavioral pathways could further clarify how these interventions exert their effects. Research targeting specific subpopulations, including youth, LGBTQ+ individuals, and those averse to faith-framed content, is also needed to evaluate cultural acceptability, optimize intervention tailoring, and identify potential differences in treatment response.

## Conclusions

This review finds that brief, structured prayer practices of about one minute are associated with lower cravings, improved coping, and better emotional regulation in addiction recovery. These practices were used across varied settings and personal beliefs, were often incorporated into daily routines, and were described as meaningful and easy to use. Their low cost and simple format suggest high feasibility, particularly in settings with limited access to healthcare.

These associations should not be interpreted as causal, given variability in study designs, reliance on self-report, and limited long-term follow-up. Further randomized trials and mechanism-focused studies are needed to test durability, clarify how effects arise, and refine delivery for diverse populations. If supported by stronger evidence, brief prayer practices could serve as an optional, patient-preferred adjunct to established treatments, not a substitute, within comprehensive, person-centered care.

## Appendices

Database search strategy and Boolean strings 

A comprehensive search was conducted in June 2025 across five databases: PubMed, PsycINFO, CINAHL, Scopus/Web of Science, and Google Scholar. Boolean logic was employed to identify studies evaluating short prayer-based interventions on addiction-related outcomes. Filters for English language, human studies, and peer-reviewed articles were applied where available.

**Table 4:** Database Search Strategy and Boolean Strings

1. PubMed
URL: https://pubmed.ncbi.nlm.nih.gov
Search String:
("serenity prayer" OR "short prayer" OR "brief prayer" OR "daily prayer" OR "spiritual affirmation" OR "religious invocation" OR "faith-based mantra") AND ("substance use disorder" OR "SUD" OR "opioid use disorder" OR "alcohol use disorder" OR "addiction" OR "12-step program" OR "AA" OR "NA" OR "Celebrate Recovery") AND ("treatment outcome" OR "recovery" OR "relapse" OR "craving" OR "sobriety" OR "abstinence" OR "coping" OR "emotion regulation")
Filters Applied:
English, Humans, Last 20 years
2. PsycINFO (via EBSCOhost)
Search String:
TI,AB("serenity prayer" OR "short prayer" OR "brief prayer" OR "daily prayer" OR "religious mantra" OR "spiritual invocation") AND TI,AB("substance use disorder" OR "addiction" OR "recovery" OR "12-step program" OR "AA" OR "NA") AND TI,AB("relapse" OR "craving" OR "coping" OR "abstinence" OR "emotional regulation" OR "treatment effectiveness")
Filters Applied:
Peer-reviewed, English, Human, Journal Articles
3. CINAHL (via EBSCOhost)
Search String:
("serenity prayer" OR "short prayer" OR "brief prayer" OR "religious mantra" OR "spiritual practice") AND ("addiction" OR "substance use disorder" OR "recovery" OR "12-step program" OR "alcoholism" OR "opioid use") AND ("craving" OR "relapse" OR "emotional regulation" OR "sobriety" OR "treatment outcome")
Filters Applied:
English, Peer-reviewed, Humans, Nursing or Allied Health Journals
4. Scopus / Web of Science
Search String:
Scopus: TITLE-ABS-KEY("serenity prayer" OR "short prayer" OR "brief prayer" OR "religious phrase" OR "daily prayer") AND TITLE-ABS-KEY("addiction" OR "substance use disorder" OR "opioid use disorder" OR "recovery" OR "12-step" OR "faith-based recovery") AND TITLE-ABS-KEY("treatment outcome" OR "relapse" OR "craving" OR "coping" OR "sobriety")
Web of Science: TS=("serenity prayer" OR "brief prayer" OR "religious invocation" OR "short spiritual practice") AND TS=("substance use disorder" OR "addiction" OR "opioid" OR "12-step" OR "faith-based recovery") AND TS=("recovery" OR "treatment outcome" OR "craving" OR "sobriety")
Filters Applied:
English, Last 20 years, Document Type: Article or Review
5. Google Scholar
URL: https://scholar.google.com
Search String:
"serenity prayer" OR "short prayer" AND "substance use disorder" OR "addiction" OR "12-step program" AND "recovery" OR "craving" OR "relapse"
Filters Applied:
Use quotes for exact phrases; Filter by year (e.g., since 2010); Prioritize peer-reviewed PDFs and reputable sources (.org, .edu)
